# Anti-MuSK-Positive Myasthenic Crisis in a 7-Year-Old Female

**DOI:** 10.1155/2017/8762302

**Published:** 2017-04-30

**Authors:** Harrison J. Matthews, Apisadaporn Thambundit, Brandon R. Allen

**Affiliations:** ^1^Department of Emergency Medicine, UF Health Shands Hospital, Gainesville, FL, USA; ^2^Department of Pediatrics, UF Health Shands Hospital, Gainesville, FL, USA

## Abstract

A seven-year-old African American female with anti-MuSK-positive Juvenile Myasthenia Gravis collapsed while at school from progressively worsening weakness and dyspnea. On initial emergency department presentation, she required 15 liters per minute of supplemental oxygen to maintain oxygen saturation above 92%. Initial pulmonary function tests and venous blood gas led to the decision to place her on noninvasive positive pressure ventilation (NPPV) with BiPAP in the emergency department. Due to worsening hypercarbia, she later required mechanical intubation in the PICU and underwent IVIG therapy followed by plasmapheresis in order to achieve a stable discharge from the hospital. A respiratory virus panel PCR was positive for influenza A, influenza B, and rhinovirus, likely precipitating the respiratory failure and myasthenic crisis in this seven-year-old patient. Given the rarity of this condition, this case report is to provide further education to the clinician managing severe, prepubertal Juvenile Myasthenia Gravis and myasthenic crisis.

## 1. Introduction

Myasthenia Gravis (MG) is an autoimmune disease characterized by the decreased availability of acetylcholine receptors at the neuromuscular junction, resulting in fluctuating muscle weakness and fatigability. Juvenile Myasthenia Gravis (JMG), classified as MG diagnosed before the age of 19, exhibits both similarities and differences from the adult form [1]. Initial diagnosis can be complicated by the atypical presentation of JMG and also with the multitude of other neuromuscular disorders on the differential diagnosis including a congenital myasthenia syndrome, mitochondrial myopathy, or Guillain-Barre syndrome.

Diagnosis can be made with the presence of antibodies at the neuromuscular junction. Presence of antibodies to the nicotinic acetylcholine receptor (AChR) is most common, with a positive result in 80% of adults with generalized Myasthenia Gravis [1]. However, the presence of antibodies to muscle-specific kinase (MuSK), a neuromuscular junction protein, also confirms a diagnosis of MG. Termed anti-MuSK-positive MG, this subgroup is rare in children and is associated with more severe disease, weakness of the facial and bulbar muscles, and more frequent respiratory crisis. Interestingly, African American patients with JMG exhibit a lower rate of remission, with higher prevalence in females [2].

Managing respiratory crisis in prepubertal MG is not well documented, likely due to the higher correlation of isolated ocular muscle weakness in this population and higher rates of spontaneous or therapy induced remission [3]. The seven-year-old female patient presented in this case report had failed initial treatment with anticholinesterase and had undergone monthly intravenous immunoglobulin (IVIG) for nearly one year when she developed myasthenic crisis necessitating mechanical ventilation.

## 2. Case Presentation

A seven-year-old African American female, with known anti-MuSK-positive JMG, arrived to the pediatric emergency department after she had collapsed at school. Her clinical course began about 15 months prior, with lower extremity pain while running, abnormal gait, decreased strength, and facial palsy. She later developed acute ophthalmoplegia with bilateral ptosis, at which time she was diagnosed using electromyography (EMG) and the Tensilon test. Initial AChR antibodies (binding, blocking, modulating) returned negative; however, MuSK antibody titers returned positive with titer levels of 1 : 5210. She failed anticholinesterase therapy with pyridostigmine and decision was made to begin IVIG therapy one month after diagnosis due to severe presentation. She continued monthly IVIG therapy due to good clinical response without other daily medication. She had no prior episodes of respiratory distress or failure, and, during prior admissions, she underwent serial negative inspiratory force (NIF) and forced vital capacity (FVC) measurements by respiratory therapy (RT).

The patient was scheduled for IVIG infusion on the day of her ED presentation but unfortunately failed to present for the appointment. A concern for nonadherence was documented throughout the medical record. The mother noted that she had cough and congestion that morning, but, otherwise, she was in her normal state of health without associated fever or muscle aches. While at school, the counselor found her to have bilateral ptosis, shortness of breath, and progressively increasing weakness, consequently leading to collapse. She remained awake and alert throughout the entire event. EMS was called, and on arrival she was noted to have an oxygen saturation (SpO2) in the 70s for which she required supplemental oxygen. She arrived to the pediatric emergency department on 15 liters of oxygen via mask and maintained SpO2 of 94–97%. When off supplemental oxygen, she was hypoxic with SpO2 of 83%. She arrived in moderate respiratory distress; the patient was only able to speak in short sentences, exhibited decreased air movement and shallow breathing, and had a respiratory rate of 33 breaths per minute. Pediatric ICU and neurology were immediately contacted due to concern of impending respiratory failure.

In order to assess inspiratory muscle function, NIF measurements were obtained by RT, with the following results: −20 cm mmH2O and −24 cm mmH2O. For this reason, a bedside venous blood gas (VBG) was drawn, notable for a significantly decreased pH of 7.28, pCO2 of 77 mmHg, and an oxygen saturation of 52%. Due to hypercarbic respiratory acidosis, tachypnea, and hypoxia, she was immediately placed on noninvasive positive pressure ventilation (NIPPV) (Philips Respironics V60) with the following settings: inspiratory positive airway pressure (IPAP) of 14 cm H2O, expiratory positive airway pressure (EPAP) of 12 cm H2O, respiratory rate of 15, and FiO2 of 100%. The patient responded well with improved respiratory effort and clear breath sounds bilaterally. IVIG was started in the emergency department and patient was successfully transported to the ICU within 45 minutes of arrival.

She remained clinically stable on NIPPV for fourteen hours but deteriorated after PICC line placement with poor chest rise and VBG showing worsening respiratory acidosis (pH of 7.18 and pCO2 90), thus requiring intubation. She tolerated intubation well with versed and fentanyl and was placed on mechanical ventilation under volume control mode with PEEP of 8, tidal volume of 210 mL (6.6 ml/kg), and FiO2 of 25%. She remained intubated for two days prior to being transitioned to NIPPV with IPAP of 14, EPAP of 7, and FiO2 of 30%. Her strength returned to baseline after two doses of IVIG (1 g/kg/day per dose). On day 6, she was transitioned to high flow nasal cannula at 10 liters per minute and FiO2 of 25% and weaned to room air within the next 24 hours. Unfortunately, she had to be placed back on NIPPV with IPAP of 10, EPAP of 5, and FiO2 of 30% that night due to desaturation to the 70s, end tidal volume CO2 of 61.6, and VBG CO2 of 64.9. Thus, neurology recommended initiating plasmapheresis, of which she completed five rounds. She was stable on room air by the third round of plasmapheresis. Her respiratory viral panel PCR was positive for influenza A and B and rhinovirus, likely precipitating the myasthenic crisis. She was treated with Tamiflu and was discharged on day 16 of hospitalization.

## 3. Discussion

Juvenile Myasthenia Gravis is rare making up only 10 to 20% of all Myasthenia Gravis patients. There is higher prevalence in females with a 1.3–1.8 to 1 ratio [4]. Diagnosis is made based on the patient's history (weakness and fatigability of skeletal muscles), clinical examination (ocular and/or bulbar weakness), electromyography, pharmacological testing (Tensilon test), and presence of specific antibodies at the neuromuscular junction. However, diagnosis of JMG especially in prepubertal patients can pose a challenge as nonspecific symptoms can be present and antibodies are often undetectable [2].

Treatment of JMG was adopted from adult patients and includes anticholinesterase drugs, steroids, immunosuppressants, plasmapheresis, IVIG, and thymectomy. A recent international consensus guidance for management of Myasthenia Gravis recommended that young children with only ocular symptoms of MG can be treated initially with pyridostigmine and that long-term treatment with corticosteroids should be at the lowest effective dose to minimize side effects of growth failure and poor bone mineralization. Additionally, maintenance IVIG or plasmapheresis are alternatives to immunosuppressive drugs in this population. In myasthenic crisis, IVIG and plasmapheresis are appropriate therapies, though the choice between one over the other depends on the comorbidity of each patient. The authors noted that plasmapheresis is more effective than IVIG in anti-MuSK MG, which may be representative in our case since she required plasmapheresis in addition to IVIG for clinical improvement [5]. Plasmapheresis provides a safe and quick way to remove serum antibodies. Various mechanisms have been hypothesized for IVIG including downregulation of antibodies by directly binding to the antibodies, acting as a competitor to the postsynaptic receptors, and inhibiting the synthesis of the antibodies themselves [6].

Myasthenic crisis can be life-threatening and often involves critical care monitoring. It is defined as an acute neuromuscular respiratory failure due to a relative decrease in the amount of acetylcholine at the NMJ. Triggers include stress, infection, and surgery, though sometimes the etiology is idiopathic [2, 3]. It is vital to assess respiratory function and be prepared to provide support whether it is in the form of noninvasive ventilation or intubation and mechanical ventilation until the patient receives appropriate immunomodulatory therapy (plasmapheresis or IVIG). Pyridostigmine therapy should not be used in myasthenic crisis. Neurology and the critical care team should be contacted promptly for care management [3].

There is little data published on initial airway management of prepubertal patients in myasthenic crisis. The unpredictable nature of these patients' respiratory status and poorly predictive values of their initial pulmonary function tests [7] make it difficult to decide between unnecessary intubation and iatrogenic harm and intubation prior to respiratory exhaustion. In adults, many studies have shown NIV to play a role in helping to prevent intubation [8–10]. Rabinstein believes that NIV is most useful in patients with myasthenic crisis in the acute setting, especially BiPAP as it provides inspiratory pressure to overcome upper airway resistance, expiratory pressure to support muscle fatigue, and flow of oxygen. Prompt use of NIV, specifically initiation prior to hypercapnia can reverse worsening fatigue and prevent complete muscle failure. It is also a useful tool after extubation in patients recovering from a myasthenic crisis [11]. If a patient requires intubation, a nondepolarizing agent such as rocuronium and vecuronium is preferred. Nonfunctioning acetylcholine receptors are resistant to depolarizing agents like succinylcholine and paralysis is prolonged when achieved with higher doses [12]. Clinical symptoms, NIF, and blood gases are all important tools to help with management of patients with myasthenic crisis.

This case describes a 7-year-old girl with anti-AChR negative, anti-MuSK positive JMG who presented in myasthenic crisis due to an upper respiratory viral infection. She experienced ocular muscle weakness and worsening respiratory effort resulting in hypoxia and hypercapnia. She initially responded well to NIPPV and IVIG therapy but eventually required intubation and mechanical ventilation due to worsening respiratory acidosis. She regained muscle strength after plasmapheresis and was transitioned back to NIPPV prior to successfully being weaned to room air and successfully discharged. NIF and blood gases played important roles in the initial assessment and management of this case.

We hope that this case provides additional information to the little available data on initial management of a Juvenile Myasthenia Gravis patient in crisis and respiratory failure. Prompt multidisciplinary management by the pediatric ED, ICU, and neurology teams in addition to close monitoring of the patient's symptoms while considering the most effective and least invasive interventions contributed to this patient's successful treatment.
